# Phase I 270° single-incision percutaneous spinal endoscopy for decompression treatment of thoracic spinal stenosis

**DOI:** 10.1038/s41598-022-13666-4

**Published:** 2022-06-08

**Authors:** Yuefei Li, Jingwei Bi, Zhaozhong Sun, Jiabin Ren, Xin Liu, Ning Sun, Jianye Wang, Rui Li

**Affiliations:** Department of Spine, Binzhou Medical University Hospital, No. 661 Huanghe 2nd Road, Binzhou City, Shandong Province China

**Keywords:** Neuroscience, Diseases

## Abstract

This study aimed to explore the feasibility of Phase I percutaneous spinal endoscopy with a 270° single incision in the ventral and dorsal dura mater for decompression treatment of thoracic spinal stenosis (TSS). Phase I percutaneous spinal endoscopy with a two-path (posterior and posterolateral approaches) single incision with a 270° decompression was performed in four cases of TSS with compression in the ventral and dorsal dura mater. The affected intervertebral space was located during the surgery, and the ossified ligamentum flavum in the ventral and dorsal dura mater was removed via laminectomy, which formed a decompression space in the thoracic cord. Next, posterolateral transforaminal expansion and plasty were performed to remove the ventral intervertebral disk. The visual analogue scale (VAS) score, thoracic spinal cord function score of the Japanese Orthopaedic Association (JOA) (11-point method), and Oswestry Disability Index (ODI) scores were used to evaluate the clinical efficacy. No dura mater or thoracic nerve injury occurred during the surgery. The symptoms of weakness in the lower extremities improved after the surgery. The postoperative magnetic resonance imaging and computed tomography examinations showed compression removal and dura mater bulging. The postoperative VAS, JOA, and ODI scores improved compared with the preoperative scores. Two surgical trajectories, posterior and posterolateral approaches, were established by a single incision using thoracic spinal canal decompression with Phase I 270° single-incision percutaneous spinal endoscopy. The posterior approach was performed mainly by translaminar unilateral fenestration and bilateral decompression in the ventral and dorsal dura mater, whereas the posterolateral approach was performed by decompression in the ventral dura mater to the midline of the vertebrae. This surgical method could be applied as a safe and feasible minimally invasive treatment for TSS with compression on both the ventral and dorsal dura mater.

## Introduction

Thoracic spinal stenosis (TSS) is mainly caused by thoracic disk herniation (TDH) and ossification of the ligamentum flavum (OLF). The incidence of TDH is low, accounting for 0.15–4% of the population undergoing discectomy, and 75% of the lesions are located in the lower thoracic spine (T_9_–L_1_)^1–3^. TDH is classified into central, paracentral, and lateral TDH based on the compression site, and the lateral type is not common^1,4^. OLF is rare in spinal diseases and mainly occurs in East Asia^5^. It is divided into unilateral laminar OLF and bilateral laminar OLF according to the compression site. The spinal and thoracic segments have a complex anatomy and adjacent to important organs, such as the pleural cavity. Additionally, they present less tolerant thoracic cord and rib obstruction pathologically. Hence, it is challenging and risky to perform thoracic endoscopic techniques. Traditional open surgery for TSS, such as the translaminar approach, anterior pleural approach, vertebral pedicle approach, transverse costal process approach, and posterolateral pleural approach, is associated with considerable trauma, slow recovery, and many complications^6–11^. The application of percutaneous spinal endoscopy has been expanded from the treatment of cervical and lumbar vertebral diseases to that of thoracic vertebral diseases as well as from intervertebral disk herniation to spinal stenosis^12^. However, the endoscopic management of TSS with compression in the ventral and dorsal thoracic cords caused by TDH combined with OLF is difficult and has not yet been reported. This study reports the clinical data of four cases of TSS with compression in the ventral and dorsal thoracic cords treated by percutaneous spinal endoscopy.

## Preoperative preparation

Patients had a sensation of weakness on both lower extremities and gait similar to stepping on cotton. X-ray, computed tomography (CT), and magnetic resonance imaging (MRI) examinations were conducted before the surgery, suggesting thoracic intervertebral disk herniation on the ventral side of the dura mater and OLF on the dorsal side. The preoperative general conditions of the patients are shown in Table 1. Patients were informed of the treatment plan and risks. They signed an informed consent form and fasted before the surgery.

**Table 1:** General data of patients.

	Case 1	Case 2	Case 3	Case 4
Age (years)	52	56	53	63
Sex	Female	Female	Male	Male
Follow-up time (month)	15	13	16	20
Lesion segment	T_12_/L_1_	T_10/11_	T_1/2_	T_11/12_
Type of TDH	Paracentral	Paracentral	Paracentral	Central
Location of OLF	Unilateral lamina	Bilateral lamina	Unilateral lamina	Unilateral lamina
Symptom duration (month)	8	14	6	11
Chest and abdomen band sensation	–	+	–	–
Symptoms of lower limb weakness	+	+	+	+
Hypesthesia	+	+	+	+
Muscle force of affected extremities (grade)	IV	III	IV	IV
Bowel and bladder dysfunction	No	No	Yes	No

## Surgical method

**Figure 1:**
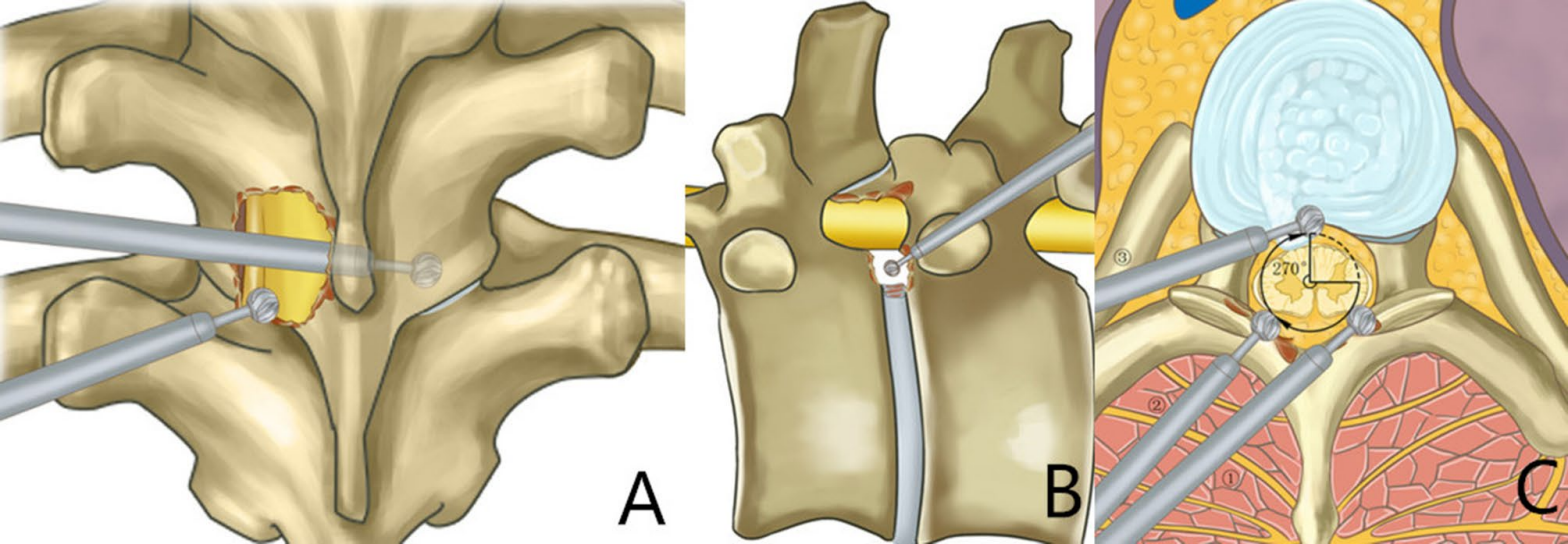
Illustration of the critical process in 270° decompression technique of spinal endoscopy. (**A**) The window of the posterior approach vertebral plate was opened. First, contralateral decompression was performed and then ipsilateral decompression was performed. (**B**) The posterolateral approach was used to remove the ventral disc and part of the vertebral bone; (**C**) The percutaneous spine endoscopic technique accomplished phase I compression in the ventral and dorsal dura mater using 270° decompression.

Patients were given general or local anaesthesia, and the prone position was adopted. With C-arm fluoroscopy, the body surface was positioned at the level of the responsible intervertebral space, and a puncture point was made 5 cm lateral to the posterior median line of the spinous process to make a longitudinal incision of 7 mm in length. A thick guide rod was used to puncture and dilate the soft tissue, with the tip located at the level of the intervertebral space and near the lateral margin of the facet joints shown by fluoroscopy. Then, a working cannula was placed, and the endoscopic system was positioned. With the endoscope, a Kirschner wire was placed in a hole of the bone surface using a drill, and fluoroscopy was conducted again to determine the lesion segment. First, the working cannula was moved medially to the dorsal side of the lamina. A drill was used to remove the lamina between the area near the medial edge of the facet joints and the base of the spinous process under endoscope visualization. The contralateral ossified ligamentum flavum was thinned and made transparent by the appropriate abduction of the cannula to or was completely removed. The dura mater was separated from the ossification, followed by the removal of the ossification of the nucleus pulposus with a pincer. After decompression, the direction of the working cannula was transferred to the same side, and the ossified ligamentum flavum was treated in the same way. Following complete decompression in the dorsal dura mater, the working cannula was moved outward to the dorsal side of the intervertebral foramen, and part of the bone on the lateral side of the facet joints and the upper edge of the pedicle in turn were removed. The intervertebral foramen was enlarged, the intervertebral space and the lateral edge of the dura mater were exposed, and the herniated intervertebral disk was explored. Then, the soft herniated disk was moved directly. For calcified disk herniation, the lateral edge of the dura mater was exposed under the endoscope, and a 5-mm incision was made laterally to the edge of the dura mater (matching the diameter of the drill). Furthermore, the drill and trephine were used to remove part of the bone and intervertebral disk tissue on the posterior wall of the adjacent superior and inferior vertebral bodies to the anterior dura mater, near the midline of the vertebral body, from outside to inside and shallow to deep. The drill was used to grind laterally to the ventral side of the posterior longitudinal ligament from deep to shallow to gradually remove the herniated and calcified intervertebral disk until collapse occurred in the compressed dura mater, confirming that decompression was completed. Finally, the working cannula was withdrawn, and the incision was sutured. The procedure is shown in Fig. 1.

## Postoperative treatment

After recovery from anaesthesia, attention was given to patients regarding the changes in sensation and muscle force in their lower limbs. Patients were given hormones, treated for dehydration, received neurotrophic drugs and had out-of-bed activity after 24 h. CT and MRI examinations were conducted again 2 days after the surgery to observe the removal of lesions in the thoracic spinal canal.

## Observational indicators

The surgical time, intraoperative bleeding volume, and complications were recorded. The visual analogue scale (VAS) score, thoracic spinal cord function score of the Japanese Orthopaedic Association (JOA) (11-point method), and Oswestry Disability Index (ODI) were determined before the surgery and 1 month, 3 months, 6 months, and 12 months after the surgery.

## Results

**Figure 2:**
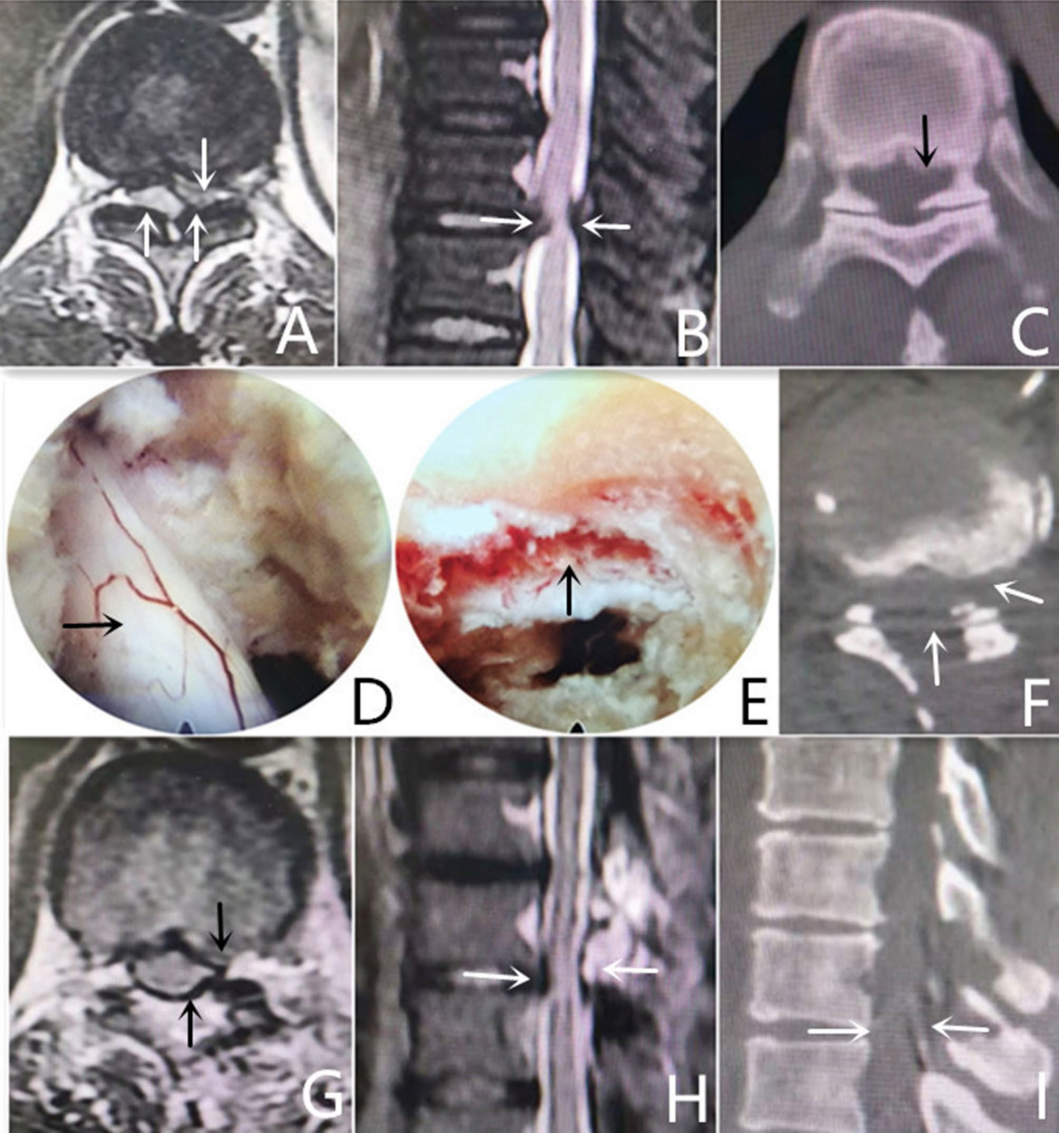
Typical cases. (**A**–**C**) Preoperative CT and MRI examinations showed T_10/11_ disk herniation on the left side with ossification of the ligamentum flavum and thoracic spinal cord degeneration in the corresponding segment. (**D**) Dura mater exposure after decompression via the posterior approach. (**E**) Dura mater margin exposure under decompression via the posterolateral approach. (**F**–**I**) Postoperative CT and MR showed that the disc herniated, the ossified ligamentum flavum was removed, and there was no compression in the dura mater.

All four patients had successful surgeries with a surgical time of 65–140 and 97.5 min on average, and the intraoperative bleeding volume was 20–45 and 30 mL on average. No nerve tears, cerebrospinal fluid leakage, or nerve and pleural cavity injuries were observed. The immediate spinal cord function improved after the surgery, without worsening symptoms of the lower limbs. The postoperative imaging of the four cases showed no compression in the intraspinal dura mater, no recurrence of disk herniation, and no stenosis or degeneration of the intervertebral space adjacent to the lesion. The VAS, JOA, and ODI scores of the four patients after the surgery significantly improved; they are shown in Table 2. Typical cases are shown in Fig. 2.

**Table 2:** Preoperative and postoperative VAS, JOA, and ODI scores of four cases.

Indicator	Follow-up	Case 1	Case 2	Case 3	Case 4
VAS (point)	Preoperation	7	6	8	7
	1 month after surgery	3	2	3	2
	3 months after surgery	2	1	3	2
	6 months after surgery	1	1	2	2
	12 months after surgery	1	1	2	2
JOA (point)	Preoperation	6	7	4	6
	1 month after surgery	9	10	8	9
	3 months after surgery	9	10	9	10
	6 months after surgery	10	10	9	10
	12 months after surgery	10	11	9	10
ODI (%)	Preoperation	60.0	56.7	70.0	61.7
	1 month after surgery	31.7	20.0	36.7	30.0
	3 months after surgery	30.0	18.3	33.3	26.7
	6 months after surgery	30.0	16.7	31.7	25.0
	12 months after surgery	30.0	16.7	31.7	21.7

## Discussion

The main causes of TSS are ossified or hypertrophic ligamentum flavum compression in the dorsal thoracic spinal cord and ventral TDH, ossification of the posterior longitudinal ligament, and hyperplastic osteophytes at the posterior margin of the vertebral body^13,14^. Isaacs^15^ first performed percutaneous spinal endoscopic posterolateral thoracic discectomy on cadavers and treated T_5/6_–T_9/10_ endoscopically without damaging the dura mater or thoracic nerve. The postoperative CT showed that this approach only required slight abrasion of the bone structure and was minimally invasive, which suggested that it could be used as a new minimally invasive option for treating TDH. Miao^16^ adopted the posterior approach of percutaneous spinal endoscopy to treat two patients with OLF, yielding impressive results. Ruetten et al.^17^ used percutaneous spinal endoscopy to treat 55 patients with TSS in T_5/6_ and lower segments, the largest sample size thus far, and proposed that percutaneous spinal endoscopy could treat all types of disk herniation except giant TDH (greater than 40% of the thoracic canal area). At present, scholars have described the percutaneous spinal endoscopic treatment of TSS with compression at a single site (ventral or dorsal; unilateral compression). However, no case of TSS with compression on either the ventral or dorsal thoracic spinal cord has been reported in the literature.

Percutaneous spinal endoscopy with a two-path (posterior and posterolateral approaches) single incision was adopted in this study. It was characterized by the establishment and sharing of only one soft tissue path and the establishment of two ossified paths. An incision 5 cm in length was made lateral to the posterior median line of the spine. The distance could be appropriately enlarged for obese patients. Surgeons often struggle with the incision length so that the surgery can be performed smoothly. As recorded in the literature, surgical incision distances using the posterior and posterolateral approaches are 2 and 6–8 cm, respectively. The study confirmed that it was easy to move the path inward or outward by 4 cm due to the loose soft tissue in the back, which could better adjust the path into a reasonable abduction angle. Performance of the conventional surgical procedures of the posterior and posterolateral approaches reduced the trauma, surgical time, and X-ray dose as well as improved the surgical efficiency. Under fluoroscopy, the working cannula was located at the level of the diseased intervertebral space, near the lateral margin of the facet joints. The ossified or hypertrophic thoracic ligamentum flavum was removed via the posterior approach, moving the cannula into the dorsal lamina. If thoracic OLF is located in the unilateral lamina, the unilateral lamina can be removed under endoscopy, and ipsilateral or contralateral spinal canal decompression can be performed. If thoracic OLF is located in the bilateral lamina, endoscopic decompression of the unilateral lamina and bilateral spinal canal is recommended without removal of the bilateral lamina. Thoracic decompression surgery is likely to cause ischaemia–reperfusion injury and to aggravate spinal cord oedema. Part of the lamina on the side with heavy compression was removed. Decompression of the dura mater was performed on the opposite side to reserve space for the thoracic spinal cord, and then the ipsilateral side with heavy compression was processed. This reduces the risk of surgery. The working cannula was moved outwardly to the dorsal side of the intervertebral foramen for the posterolateral approach. The facet joints and part of the bone at the upper edge of the pedicle were removed endoscopically, followed by enlargement and formation of the intervertebral foramen. The lesion location was exposed, and the soft herniated intervertebral disk was directly removed. Direct removal could easily cause complications such as dura mater leakage and thoracic spinal cord injury for calcified disk herniation and adhesion between the dura mater and calcified disk. Surgeons could use a drill or trephine to remove part of the bone and intervertebral disk tissue on the posterior wall of the adjacent superior and inferior vertebral bodies, with the working channel abducted by approximately 60°. The drill was used to grind the ventral side of the posterior longitudinal ligament, which exceeded the median line of the vertebral body. Using the lateral abrasion method, the calcified intervertebral disk was removed gradually using the drill until collapse occurred in the compressed dura mater, demonstrating adequate decompression. This surgical method could achieve thoracic spinal canal decompression under Phase I endoscopy with a 270° single incision via lateral and posterolateral approaches.

Phase I 270° single-incision percutaneous spinal endoscopy for TSS decompression is still technically difficult. Oltulu et al.^1^ concluded that scapular and soft tissue obstruction occurred above the T4 vertebrae, causing relative contraindications for the posterolateral approach. Liu et al.^18^ showed a scapular obstruction in the upper thoracic spine, and therefore, the posterolateral approach in this region is not recommended. At T1/2–T4/5, the working channel was blocked by the scapula and/or the soft tissue; it was located at the cervicothoracic junction near the physiological lordosis of the cervical spine, and the location of the intervertebral foramen was deep. Hence, it was difficult for the working channel to have a close abduction to the ventral side of the dura mater. However, a sufficient abduction angle of the working channel was a key step of decompression in the ventral dura mater. Although Ruetten reported a large sample size, no patients with T1/2–T4/5 TSS were observed. Hence, it was suggested that the posterolateral approach could not be performed for all types of disk herniation, although giant TDH was excluded. T1/2–T4/5 paracentral soft herniation was directly removed. However, the posterolateral approach could not provide enough abduction angle to establish a working channel for central soft or calcified herniation (contralateral edge of the calcified disk to be treated), which was a relative contraindication for endoscopic surgery. In this study, the main limitation was the small sample size. Therefore, the sample size should be further increased, and follow-up should be performed for long-term efficacy.

### Ethics approval statement

All experimental protocols were approved by the ethics committee of Binzhou Medical University Hospital.
